# Supplementary material for: Phasing single-molecule nano-NOMe-seq reveals chromatin state heterogeneity in the context of transcription and long-range interactions
Source: bioRxiv. 2025 Sep 11:2025.09.08.674887. Originally published 2025 Sep 9. Preprint. [Version 2] doi: 10.1101/2025.09.08.674887 (PMC12439907; doi:10.1101/2025.09.08.674887)
Supplement: 1 [file NIHPP2025.09.08.674887V2-supplement-1.pdf]

bioRxiv preprint doi: https://doi.org/10.1101/2025.09.08.674887; this version posted September 11, 2025. The copyright holder for this preprint (which was not certified by peer review) is the author/funder, who has granted bioRxiv a license to display the preprint in perpetuity. It is made available under aCC-BY 4.0 International license.

SUPPLEMENTARY INFORMATION

Supplemental Figures

Figure S1. Clustered-based phasing of the Nano-NOMe-seq single molecules. Cartoon depicting the agglomerative hierarchical clustering (**A**), the Hungarian algorithm (**B**) and (**C**) the Hamming distance used in the clustered based phasing pipeline. This figure relates to **Figure 2**.

Figure S2. Cluster-based phasing of nano-NOMe-seq data reveals CTCF-independent Sox2 transcription. Profiles of mean methylation across all 9 clusters (excluding the fully methylated and unmethylated clusters, C1 and C9) and studied windows shown in **Figure 4A**. This figure relates to **Figure 4**.

Figure S3. Cluster-based phasing of nano-NOMe-seq data reveals long range multiway CTCF dependent loops at the *Sox2* locus. A. Profiles of mean methylation across all 7 clusters (excluding the fully methylated and unmethylated clusters, C1 and C7) and studied windows shown in **Figure 5A**. This figure relates to **Figure 5**.

Figure S4. Cluster-based phasing of nano-NOMe-seq data reveals long range high-order multiway CTCF independent loops between micro-compartments at the *Klf1* locus. A. Profiles of mean methylation across all 8 clusters (excluding the fully methylated and unmethylated clusters, C8 and C9) and studied windows shown in **Figure 7A**. This figure relates to **Figure 7**.

bioRxiv preprint doi: https://doi.org/10.1101/2025.09.08.674887; this version posted September 11, 2025. The copyright holder for this preprint (which was not certified by peer review) is the author/funder, who has granted bioRxiv a license to display the preprint in perpetuity. It is made available under aCC-BY 4.0 International license.

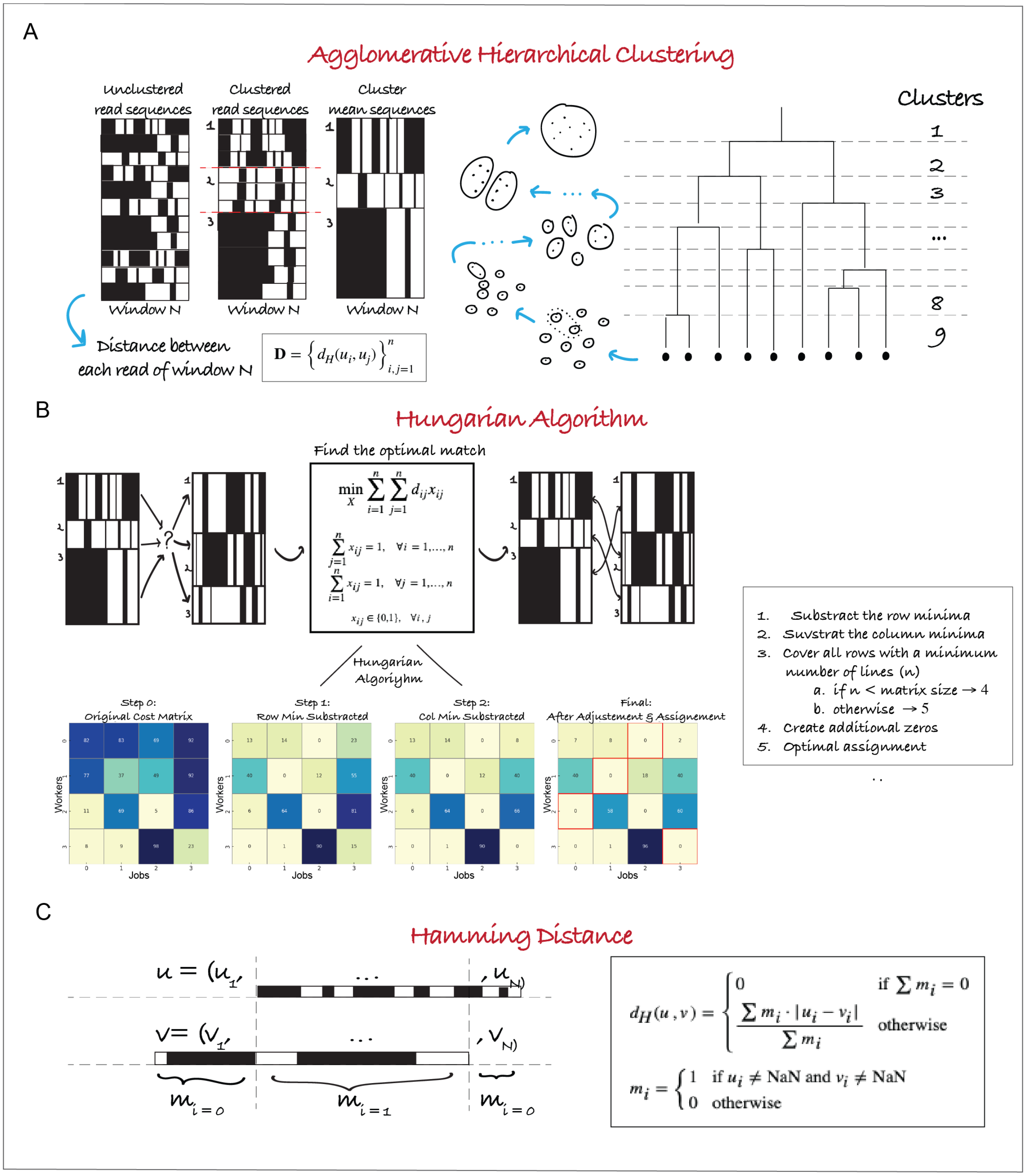

Figure S1. Clustered-based phasing of the Nano-NOMe-seq single molecules. Cartoon depicting the agglomerative hierarchical clustering (**A**), the Hungarian algorithm (**B**) and (**C**) the Hamming distance used in the clustered based phasing pipeline. This figure relates to **Figure 2**.

bioRxiv preprint doi: https://doi.org/10.1101/2025.09.08.674887; this version posted September 11, 2025. The copyright holder for this preprint (which was not certified by peer review) is the author/funder, who has granted bioRxiv a license to display the preprint in perpetuity. It is made available under aCC-BY 4.0 International license.

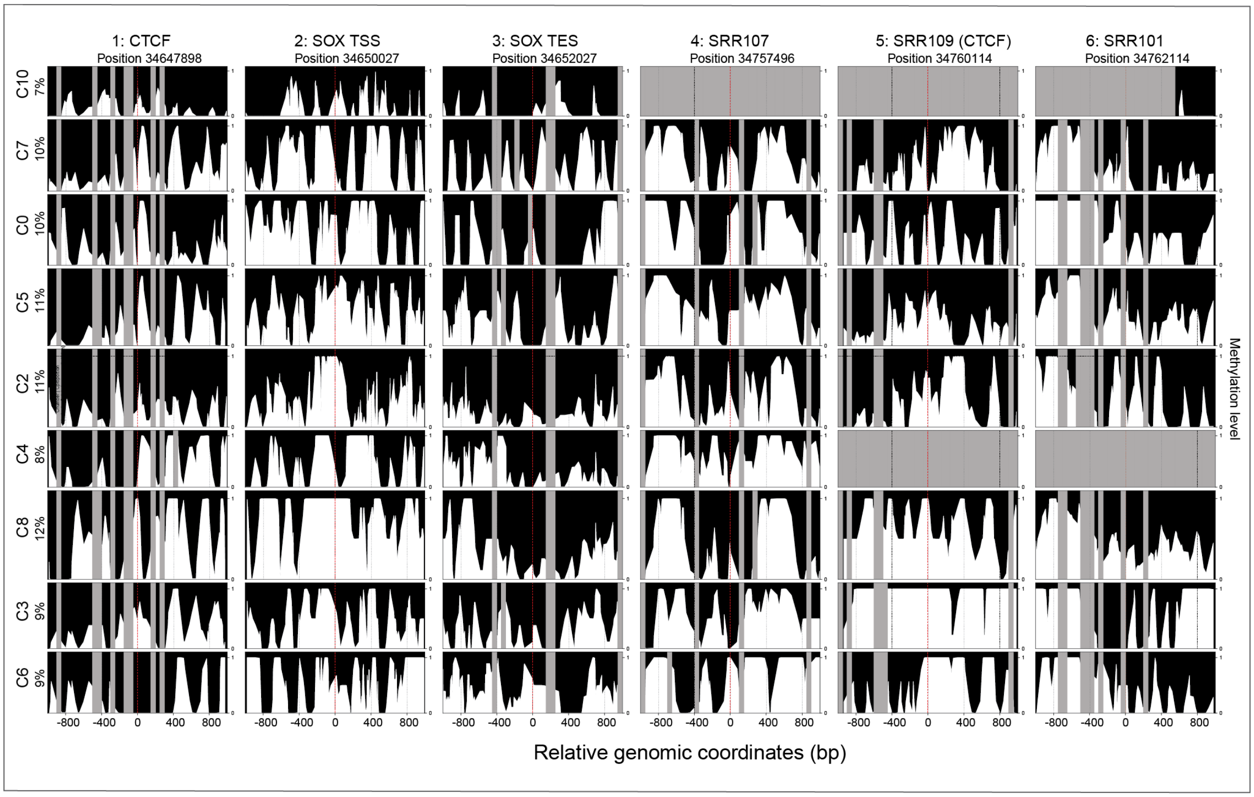

Figure S2. Cluster-based phasing of nano-NOMe-seq data reveals CTCF-independent Sox2 transcription. Profiles of mean methylation across all 9 clusters (excluding the fully methylated and unmethylated clusters, C1 and C9) and studied windows shown in **Figure 4A**. This figure relates to **Figure 4**.

bioRxiv preprint doi: https://doi.org/10.1101/2025.09.08.674887; this version posted September 11, 2025. The copyright holder for this preprint (which was not certified by peer review) is the author/funder, who has granted bioRxiv a license to display the preprint in perpetuity. It is made available under aCC-BY 4.0 International license.

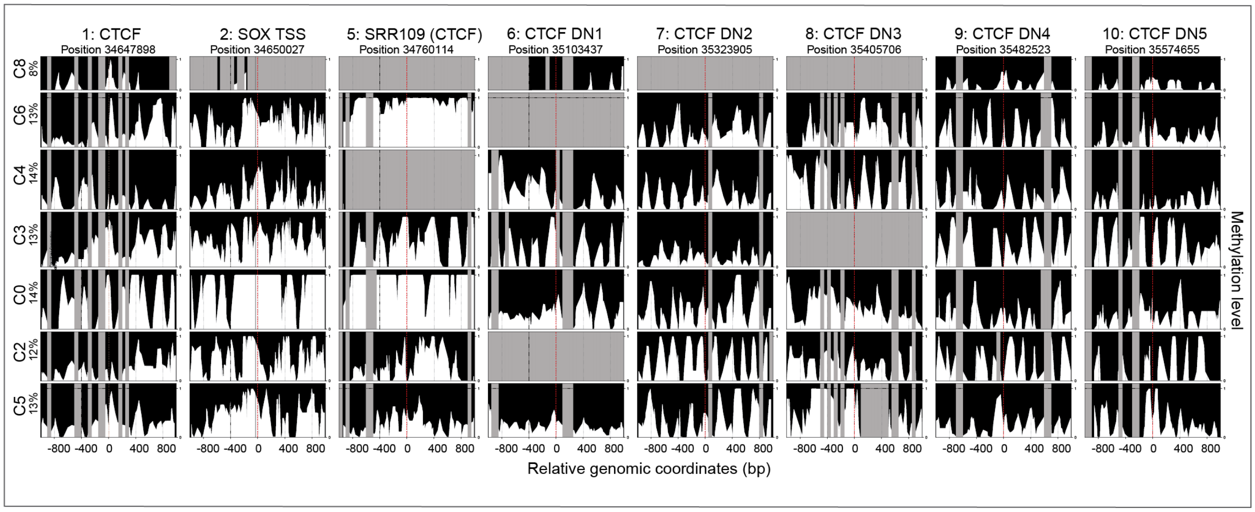

Figure S3. Cluster-based phasing of nano-NOMe-seq data reveals long range multiway CTCF dependent loops at the *Sox2* locus. A. Profiles of mean methylation across all 7 clusters (excluding the fully methylated and unmethylated clusters, C1 and C7) and studied windows shown in **Figure 5A**. This figure relates to **Figure 5**.

bioRxiv preprint doi: https://doi.org/10.1101/2025.09.08.674887; this version posted September 11, 2025. The copyright holder for this preprint (which was not certified by peer review) is the author/funder, who has granted bioRxiv a license to display the preprint in perpetuity. It is made available under aCC-BY 4.0 International license.

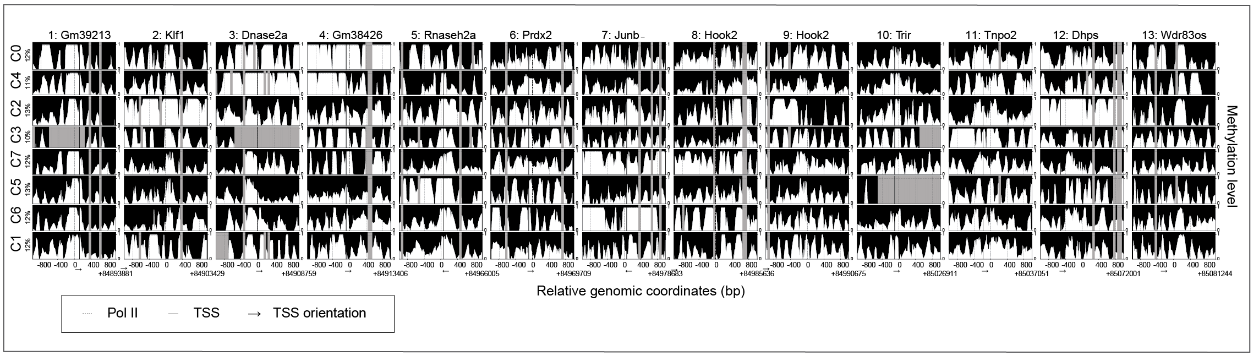

Figure S4. Cluster-based phasing of nano-NOMe-seq data reveals long range high-order multiway CTCF independent loops between micro-compartments at the *Klf1* locus. A. Profiles of mean methylation across all 8 clusters (excluding the fully methylated and unmethylated clusters, C8 and C9) and studied windows shown in **Figure 7A**. This figure relates to **Figure 7**.
